# Online CBT life skills programme for low mood and anxiety: study protocol for a pilot randomized controlled trial

**DOI:** 10.1186/s13063-016-1336-y

**Published:** 2016-04-27

**Authors:** Christopher Williams, Carrie-Anne McClay, Rebeca Martinez, Jill Morrison, Caroline Haig, Ray Jones, Paul Farrand

**Affiliations:** Institute of Health and Wellbeing, University of Glasgow, Gartnavel Royal Hospital, 1st Floor Administration Building, 1055 Great Western Road, Glasgow City, G12 0XH Scotland UK; Mersey Care NHS Trust, Ferndale Unit, University Hospital Aintree, Lower Lane, Liverpool, L9 7AL England UK; General Practice & Primary Care, Institute of Health and Wellbeing, University of Glasgow, 1 Horselethill Road, Glasgow, G12 9LX Scotland UK; Robertson Centre for Biostatistics, Boyd Orr Building, University of Glasgow, Glasgow, G12 8QQ Scotland UK; Faculty of Health and Human Sciences, University of Plymouth, Drake Circus, Plymouth, Devon, PL4 8AA England UK; Clinical Education, Development and Research (CEDAR); Psychology, University of Exeter, Washington Singer Laboratories, Perry Road, Exeter, EX4 4QG UK

**Keywords:** Anxiety, Bibliotherapy, computerized CBT, Depression, Guided self-help, Life skills, Low mood, Online, Psychotherapy, RCT, Treatment gap

## Abstract

**Background:**

Low mood is a common mental health problem with significant health consequences. Studies have shown that cognitive behavioural therapy (CBT) is an effective treatment for low mood and anxiety when delivered one-to-one by an expert practitioner. However, access to this talking therapy is often limited and waiting lists can be long, although a range of low-intensity interventions that can increase access to services are available. These include guided self-help materials delivered via books, classes and online packages. This project aims to pilot a randomized controlled trial of an online CBT-based life skills course with community-based individuals experiencing low mood and anxiety.

**Methods:**

Individuals with elevated symptoms of depression will be recruited directly from the community via online and newspaper advertisements. Participants will be remotely randomized to receive either immediate access or delayed access to the Living Life to the Full guided online CBT-based life skills package, with telephone or email support provided whilst they use the online intervention. The primary end point will be at 3 months post-randomization, at which point the delayed-access group will be offered the intervention. Levels of depression, anxiety, social functioning and satisfaction will be assessed.

**Discussion:**

This pilot study will test the trial design, and ability to recruit and deliver the intervention. Drop-out rates will be assessed and the completion and acceptability of the package will be investigated. The study will also inform a sample size power calculation for a subsequent substantive randomized controlled trial.

**Trial registration:**

ISRCTN ISRCTN12890709

## Background

Depression is common, with an estimated 350 million people experiencing this mood disorder worldwide [1]. It has a significant impact on health, wellbeing, employment status and social functioning and is the primary cause of disability globally [1]. Various interventions are available for the treatment of depression, such as antidepressant medication and talking therapies, including low-intensity guided self-help interventions as well as high-intensity interventions, often delivered face-to-face, such as individual high-intensity cognitive behavioural therapy (CBT), interpersonal therapy and behavioural activation [2].

However, only between 10 % and 50 % of individuals receive appropriate treatment for their depression within health services [1, 3]. This treatment gap may depend on a number of factors, including non-recognition of the problem (by the individual or healthcare workers), limited knowledge about treatment options and a lack of mental health resources, resulting in long waiting lists. A key aim of the WHO mental health Gap Action Programme is to increase access to psychological therapies via non-expert support or healthcare workers. This could be achieved through the application of a low-intensity CBT guided self-help approach, delivered via bibliotherapy or online in the form of computerized CBT.

Various free and licensed computerized CBT packages are available, including ‘Moodgym’, ‘Beating the Blues’ and ‘Living Life to the Full’ (LLTTF). Studies show varying results regarding effectiveness and adherence to online interventions [4, 5]. A recent meta-review of the effectiveness of computerized CBT for depression found insufficient evidence to indicate which packages achieve better outcomes, indicating a need for further randomized controlled trials (RCTs) of computerized CBT to establish effectiveness [6].

The LLTTF course puts bibliotherapy and life skills training at the centre of the course, providing various ways of completing the course (reading, audiovisual modules, video), presenting a blended learning approach. The resources, which address key aspects of low mood and stress, take the form of classes [7, 8, Williams, McClay, Matthews, Haig, McConnachie, Morrison. A randomosied controlled trial of a community based group guided self-help intervention for low mood and stress. Submitted], printed books [9] and e-books, in addition to the online course. Importantly, the online version of the LLTTF intervention adheres to the key NICE recommendations regarding computerized CBT packages in terms of content, number of modules and the support element provided [2]. A previous non-randomized feasibility study suggests that the online LLTTF course helps improve mood and anxiety; this study compared LLTTF, Beating the Blues and a book-based bibliotherapy intervention, demonstrating equivalent significant improvements across the three interventions [10]. However, further research is needed regarding the efficacy of LLTTF in its online format as to date there have been no adequately powered RCTs of the LLTTF package. Also, following earlier evaluations of LLTTF, the intervention has been refined in three key ways: (1) content has been updated; (2) the delivery platform (website) has been enhanced to add planning and reminder components, alongside the option to read the online linked books and (3) automated support emails as well as a structured support algorithm have been developed [11]. The extent and timing of support is known to improve outcomes in online treatments for depression, with maximal response (a Cohen’s *d* of 0.76) when support is offered both before and during treatment, compared with 0.21 when there is no therapist support before or during treatment, 0.44 when there is therapist contact before treatment, and 0.58 when there is therapist contact during treatment only [12]. We have used the conservative estimate of effect of 0.58 to provide our sample size estimate.

### Aims

In this pilot study, the primary aim will be to investigate take-up, drop-out and completion rates of the online course and the completion rates for data collection.

Secondary outcomes will be mood ratings at 3 months. We will use changes in the Patient Health Questionnaire 9 [13] score to provide data relating to the effect of the intervention on depression levels. This will give an indication of efficacy, together with the drop-out and retention rates.

### Research questions

#### Primary question

Is the study design feasible? Is it possible to recruit from the community, remotely randomize participants, deliver the online intervention with telephone or email support and collect data at baseline and 3 months after randomization?

#### Secondary questions

1. To what extent will participants demonstrate fidelity to the online intervention?
2. Is the LLTTF online package acceptable to participants?
3. Can we confirm our ability to recruit, and achieve the required sample size in the future substantive study?

## Methods

### Overview

The study will be a parallel, two-arm pilot RCT with a 50:50 allocation ratio across the two groups. Participants will be remotely randomized to receive the online intervention immediately (immediate-access group) or after a 3 month delay following randomization (delayed-access group). The delayed-access group will serve as the control at the primary outcome point. Ethical approval has been granted by the College of Medical, Veterinary and Life Sciences Ethics Committee for Non-Clinical Research Involving Human Subjects, University of Glasgow (Ref. 200140159).

### Participants

#### Procedure for recruitment

People with clinical levels of depressive or anxiety symptoms will be recruited via a number of community-based methods (newspaper advertisements in the *Metro* and local papers, advertisement through the Action on Depression charity, including their websites, a phone support line, newsletters and local groups), supplemented by posters, and options such as Google advertisements. No participants will be recruited directly via the NHS and we will recruit exclusively from the UK. Informed consent will be collected from all potential participants prior to entry into the study.

#### Inclusion criteria

- Aged 18 or over.
- Living in the UK.
- Ability to understand written and spoken English language.
- Regular access to a computer, smart phone or tablet with audio and broadband connection.
- Score of 10 or more on the Patient Health Questionnaire 9.

#### Exclusion criteria

- High rating of suicidality (i.e., scoring 2 or 3 on item 9 of the Patient Health Questionnaire 9).
- Currently receiving any psychological intervention, such as counselling or psychotherapy.
- New or altered dose of antidepressant in the past month.
- Taking part in any other research projects.

#### Sample size

We aim to recruit up to 50 participants, consistent with the widely accepted sample size of 30–50 participants for pilot studies [14]. We have taken an estimated effect size of 0.58 to represent a typical response rate to supported or guided online interventions, such as that tested in the proposed study [12]. With a power of 0.58 and a significance level of 0.05, 48 patients per group (total *n* = 96) would be required. Therefore, recruiting 30–50 patients in this pilot trial should be sufficient to enable us to determine whether we can recruit and randomize sufficiently, as well as gauging our ability to retain participants and record follow-up data.

Drop-out rates in internet studies can vary from 1 % to 50 %. We are also planning to record suggested predictors (severity, chronicity and treatment length) [15].

### Randomization

Participants will complete initial questionnaires, and those that are eligible and have consented will then be remotely randomized to either an immediate-access or delayed-access group by the Robertson Centre for Biostatistics. Participants’ ID numbers will be passed to a separate researcher who will use the randomization function in Excel to assign participants remotely to the immediate immediate-access or delayed-access group. As this is a pilot study, we will not stratify for any variables during randomization. The randomization will be done remotely by a researcher who will not be involved in the final data analysis. After randomization, the two groups of participants will be followed up in exactly the same way. Those in the immediate-access group will be given an access code and link to the LLTTF website, including instructions on using the resources. Those in the delayed-access group will be told that they will be contacted again in 3 months; the access code will be sent once follow-up data have been received.

### Intervention

The free-access online LLTTF course consists of eight modules that teach a range of CBT-based life skills.

The eight sessions are as follows:

- Session 1: ‘Why do I feel so bad?’ – An introduction to the CBT model and the ‘vicious cycle’ of low mood that aims to help participants understand why they feel the way they do and how their thoughts, feelings, physical symptoms and behaviour are linked.
- Session 2: ‘I can’t be bothered doing anything’ – This module focuses on the impact of reduced activity. Users are encouraged to consider the things that they have stopped or reduced doing as a result of their low mood and make a plan to re-establish these activities in order to improve their mood.
- Session 3: ‘Why does everything always go wrong?’ teaches skills to help tackle negative thinking, including how to label unhelpful thoughts, identify negative thinking patterns and to turn these thoughts around to create more helpful ones.
- Session 4: ‘I’m not good enough’ – This session teaches how self-confidence is developed and teaches confidence-building techniques.
- Session 5: ‘How to fix almost everything’ – A problem-solving approach using an ‘easy four-step plan’ to help break down problems into smaller parts in order to overcome challenges.
- Session 6: ‘The things you do that mess you up’ – This session addresses unhelpful behaviours that may be worsening mood. Participants are encouraged to recognize problem behaviours such as isolating themselves, or drinking or smoking too much, and then create a plan for reducing them.
- Session 7: ‘Are you strong enough to keep your temper?’ – Participants learn to recognize the things that cause them to feel irritable or angry and the early warning signs they experience when they start to feel angry. They then learn techniques for better managing their anger to help them react differently to challenging people and situations.
- Session 8: ‘10 things you can do to feel happier straight away’ – The final module teaches key lifestyle choices that can improve mood, including healthy eating, exercise and closeness with others.

Additional course worksheets, video and audio (relaxation) resources supplement the main modules. These are available on the intervention website.

#### NICE specifications

The NICE guidelines [2] for the treatment of adults with depression advise on the content of online self-help packages. These guidelines state that such interventions should:

1. **Contain age-appropriate written resources** – The LLTTF materials use everyday language and avoid technical CBT terms. Worksheets are colourful and often include illustrations, and can be downloaded free of charge from the website. The linked module books can be either purchased from bookshops or online, or borrowed from many libraries.
2. **Contain six to eight sessions to be completed over 9–12 weeks** – The LLTTF package contains eight sessions, of which the first four sessions are considered the ‘core’ content (formulation, behavioural activation, thinking and confidence, which brings together thinking and behaviour changes).
3. **Have tasks to be completed between sessions (homework)** – A planner and review sheet is used in each module to encourage participants to plan the skills that they will practise or the advice that they will follow in the coming week.
4. **Include thought and behaviour change and monitoring** – The LLTTF package addresses helpful and unhelpful behaviour, increasing activity and a module focused on monitoring and changing unhelpful thinking patterns.
5. **Have a support element, including monitoring and reviewing progress** – Support in the package includes weekly reminders (automated emails) plus support sessions with a support worker delivered weekly via email or telephone. The amount of support time allocated can be varied to reflect the complexity of the needs of the person being supported [16]. The support is protocol driven [11] and focuses on encouraging use and application of the intervention. It is designed to encourage an individualized plan to be made at the end of each session using a ‘plan, do, review’ structure (worksheets are completed), with a focus on participants applying the skills and techniques that they have learned and making changes in their lives; using the materials to support them in doing this. To assess fidelity to the support model in the study, copies of all email supports and recordings of any phone support contacts will be made (with consent) to enable a sample of 20 (5 %) of the support sessions to be evaluated using a previously used adherence and competency checklist [11].

### Risk management

A potential risk to participants is that they might experience an increase in their feelings of low mood or anxiety during the study.

Such risks will be addressed in several ways:

1. Active suicidal risk is included in the exclusion criteria at the screening stage. Once recruited, any participant who is found to have active risk will be signposted to support through a general practitioner, the NHS 111 freephone service or the Samaritans helpline.
2. The study website includes details of sources of urgent help.
3. The trained support workers (staff or volunteers from the Action on Depression charity) are experienced in working with such depressed or anxious people and will be able to assist any individuals who may need additional help during the study by providing information regarding how to access alternative forms of support, and the appropriate action to be taken if the participants feel they need extra help.

### Follow-up data collection

The primary outcome point will be at 3 months after randomization. Given that the focus of this pilot study is on assessing the feasibility of the trial design, no additional follow-up points will be examined. However, depending on the results of this pilot study, progression to a future Phase III definitive RCT will include follow-up to 12 months. This research plan follows Medical Research Council guidance for evaluating complex interventions [17]. In the future substantive study, we will also plan to collect data at 6 months, and possibly one year, to record the sustainability of any improvements. Since the current study is a small pilot study, we are not able to test data collection at these longer time points.

Baseline measures include age, sex, ethnicity, mood, social function, and past and current sources of support. After 3 months, all randomized participants will be invited to repeat mood and social functioning questionnaires. Use of the intervention (using site login statistics) and satisfaction in the immediate-access group will be assessed and the collection of data for an economic analysis will be piloted (these data will not be analyzed). We will also assess contamination regarding other support received and whether anyone in the delayed-access group has accessed the LLTTF course or other similar resources, or otherwise had access to CBT.

### Outcome measures

The key outcomes of the study will be the success of recruitment, retention (a minimum of 75 % follow-up rate), acceptability (as measured by drop-out rates and a standardized questionnaire) and ability to gather data.

The measures used to assess the impact on mood and social functioning, as well as satisfaction with the intervention, are described next.

#### Patient Health Questionnaire 9

This is an open-access mood rating questionnaire consisting of nine questions mirroring DSM-IV depression diagnostic criteria and each rated from 0 to 3, giving a maximum score of 27 [13]. Cut-off scores are used to label depression severity as:

- 0–4, minimal depression
- 5–9, mild depression
- 10–14, moderate depression
- 15–19, moderately severe depression
- 20–27, severe depression

#### Generalized Anxiety Disorder 7

This is a seven-item questionnaire focusing on symptoms of anxiety [18]. Each item is rated according to the frequency of the described problem in the past 2 weeks. The responses are scored as follows: 0 = ‘not at all’, 1 = ‘several days’, 2 = ‘more than half the days’, 3 = ‘nearly every day’. There is a maximum score of 21. Scores of 0–5 on this measure indicate mild anxiety; 6–10, moderate anxiety; 11–15, moderately severe anxiety and 15–21, severe anxiety.

#### Work and Social Adjustment Scale

The Work and Social Adjustment Scale [19] assesses social functioning. It is a five-point questionnaire and addresses issues relating to an individual’s everyday life and functioning and the impact of the individual’s mood disorder on these areas. Responses are given on a scale of 0 to 8, with higher scores indicating a higher level of impairment. Those scoring over 20 are likely to have significant problems in their social functioning.

#### The Client Satisfaction Questionnaire

This is an eight-item questionnaire, rated using a four-point Likert scale [20]. It will be administered after the intervention to the immediate-access group only as a measure of satisfaction. Scores range from 8 to 32, with higher scores indicating greater satisfaction with the intervention in question. The internal consistency of the questionnaire, measured by Cronbach’s *α* coefficient, ranges from 0.83 to 0.93 [20].

### Statistical analysis

Recruitment, uptake and adherence to intervention will be summarized and presented as percentages. Baseline characteristics will be summarized for all participants as well as for the immediate-access and delayed-access groups separately. Two-sample *t* tests or Mann–Whitney tests, depending on distributions, and Fisher’s exact tests will be used to compare the two treatment groups and confirm that participants had been effectively randomized.

To assess efficacy, appropriate linear models will be used, using an intention-to-treat approach. Models will be adjusted for age, sex and antidepressant use at baseline. These models will be used to estimate and test the statistical significance of the within-group change between baseline and 3 months, and the between-group differences at each time point. Rates and characteristics of drop-out will be explored. Progression criteria to a full RCT would be based on adequate recruitment, evidence of possible clinical efficacy and ability to deliver the intervention and collect research data.

## Discussion

### Importance of the project

Depression is the main contributor to the global burden of disease [1] and has a significant impact on sufferers’ lives. Despite the fact that there are various evidence-based interventions for depression [2], fewer than 50 % of those affected receive appropriate treatment for low mood [3]. Low-intensity interventions, such as online CBT courses can potentially bridge the gap between onset of depression and receiving appropriate treatment by increasing access to psychological therapies. The same LLTTF content has already been evaluated in its course (group classes) and book forms, showing significant improvements in depression, anxiety, quality of life and high levels of satisfaction [7, 9, Williams, McClay, Matthews, Haig, McConnachie, Morrison. A randomised controlled trial of a community based group guided self-help intervention for low mood and stress. Submitted]. The current pilot study will, however, represent the first RCT of the updated LLTTF online package and provide key information across a range of methodological uncertainties required for a full RCT of the LLTTF online package. These include community recruitment, uptake, delivery, adherence to the intervention and data collection, with results being used to determine the sample size needed for a substantive RCT.

### Benefits to participants

Participation within this research will allow adults who are experiencing mild to moderate symptoms of low mood or anxiety to use online CBT-based life skills and other educational modules aimed at addressing these issues. Previous research on other variants of the same course suggests that the LLTTF approach can be effective in aiding a variety of problems related to low mood and stress [7, 9, Williams, McClay, Matthews, Haig, McConnachie, Morrison. A randomised controlled trial of a community based group guided self-help intervention for low mood and stress. Submitted]. The satisfaction data will help us to gain a key insight into how online websites can be best delivered and supported in the community.

### Working with the voluntary sector

In this pilot study, the Scottish charity Action on Depression will deliver the support element of the intervention using a pre-established support protocol. Support workers from this depression charity are experienced in working with the proposed population and have experience of introducing and supporting self-help materials. They can also assist any individuals who may need further help during the study by signposting them to additional support if there is concern about a participant’s wellbeing. Action of Depression have an effective system for identifying and supporting individuals in the community and the involvement of this charity may aid in accessing hard-to-reach individuals, including young men and minority ethnic groups; as well as individuals who may prefer voluntary sector support rather than traditional treatment options offered via the NHS.

### Dissemination

We aim to disseminate the findings of this pilot study in an open-access journal and via conference presentations. The journal publication will present the study findings, with the data being used to inform a future RCT protocol and a grant application. A short summary of the findings of the study will be posted online via social media accounts. We will also disseminate findings via newsletters at www.livinglifetothefull.com, which receives around 30 million hits a year from members of the public and practitioners and our partners Action on Depression.

## Trial status

The website has been designed and tested. Recruitment is expected to begin in September 2015, with the collection of outcome data starting in December 2015.

## Abbreviations

CBT: cognitive behavioural therapy
LLTTF: ‘Living Life to the Full’
NICE: National Institute for Health and Care Excellence
RCT: randomized controlled trial
WHO: World Health Organization
